# Use of the critical-care pain observation tool and the bispectral index for the detection of pain in brain-injured patients undergoing mechanical ventilation: A STROBE-compliant observational study

**DOI:** 10.1097/MD.0000000000010985

**Published:** 2018-06-01

**Authors:** Kai Shan, Wei Cao, Yuan Yuan, Jing-Jing Hao, Xiu-Mei Sun, Xuan He, Gui-Yun Li, Yu-Mei Wang, Jian-Xin Zhou

**Affiliations:** a Emergency Department; b Department of Critical Care Medicine, Beijing Tiantan Hospital, Capital Medical University, Beijing, China.

**Keywords:** bispectral index, brain injury, critical-care pain observation tool, pain assessment

## Abstract

The assessment of pain in patients with brain injury is challenging due to impaired ability to communicate. We aimed to test the reliability and validity of the critical-care pain observation tool (CPOT) and the bispectral index (BIS) for pain detection in critically brain-injured patients.

This prospective observational study was conducted in a neurosurgical intensive care unit in a University-Affiliated Hospital. Adult brain-injured patients undergoing mechanical ventilation were enrolled. Nociceptive (endotracheal suctioning) and non-nociceptive (gentle touching) procedures were performed in a random crossover fashion. Before and immediately after the procedure, CPOT was evaluated by 2 residents and 2 chief nurses, and BIS was documented. The ability to self-report pain was also assessed. The inter-observer reliability of CPOT was analyzed. The criterion and discriminant validities of the CPOT and the BIS were tested.

During the study, we enrolled 400 brain-injured patients. The ability to self-report pain was maintained in 214 (54%) and 218 (55%) patients during suctioning and gentle touching, respectively. The intraclass correlation coefficients (95% confidence interval) for inter-observer reliability of CPOT ranged from 0.86 (0.83–0.89) to 0.93 (0.91–0.94). Using self-reported pain as the reference, the area under the receiver operating characteristic curve (95% confidence interval) was 0.84 (0.80–0.88) for CPOT and 0.76 (0.72–0.81) for BIS. When the 2 instruments were combined as either CPOT ≥2 or BIS ≥88 after the procedure, the sensitivity and specificity were 0.90 (0.85–0.93) and 0.59 (0.52–0.66), respectively; and when the 2 instruments were combined as both CPOT ≥2 and BIS ≥88, the sensitivity and specificity were 0.62 (0.55—0.68) and 0.89 (0.83–0.93). Both CPOT and BIS increased significantly after suctioning (all *P* < .001) but remained unchanged after gentle touching (*P* ranging from .06 to .14).

Our criterion and discriminant validity results supported the use of CPOT and BIS to detect pain in critically brain-injured patients. Combining use of CPOT and BIS in different ways might provide comprehensive pain assessment for different purposes.

## Abstract 2

Supplemental Digital Content is available in the text

## 1 Introduction

Pain is a major concern in the management of critically ill patients during their stay in the intensive care unit (ICU), and inadequate treatment of pain leads to significant and long-term negative physiological and psychological consequences.^[1,2]^ Brain-injured patients admitted to the ICU are also vulnerable to pain because pain-related factors, such as common painful ICU procedures,^[3,4]^ injury site irritations and immobilization,^[5,6]^ also exist in this population. Studies have shown a high prevalence of moderate-to-severe pain in brain-injured patients,^[7]^ especially in neurosurgical patients during the early postoperative period.^[8,9]^ However, pain management is far from optimized in critically brain-injured patients.^[10,11]^

Pain evaluation is the first step in the management of pain,^[12]^ and the patient's self-reported pain level has long been considered the “gold standard.”^[13]^ However, the ability to communicate may be impaired in many ICU patients due to the use of sedation and mechanical ventilation or impaired consciousness. Although current guidelines recommend that behavioral pain assessment tools should be used for pain monitoring in nonverbal critically ill adults, patients with brain injury have been excluded from this recommendation.^[1,2]^ This situation poses an additional challenge to the assessment and management of pain in brain-injured patients admitted to the ICU.

The critical-care pain observation tool (CPOT) has been demonstrated to be one of the most valid and reliable behavioral scales for the assessment of pain in nonverbal ICU patients.^[1,2,14]^ Although studies have demonstrated the potential use of CPOT in brain-injured patients,^[15–19]^ the sample sizes in those studies were limited. Apart from behavioral assessments, preliminary investigations showed that a processed electroencephalogram instrument, that is, the bispectral index (BIS), might suggest procedure-related pain in patients with and without brain injury.^[20–22]^ However, up to now, no study has been conducted to investigate the combined use of a behavioral scale and BIS for the detection of pain.

In the present study, brain-injured patients undergoing mechanical ventilation were enrolled, and nociceptive and non-nociceptive procedures were performed. The CPOT was evaluated, and the BIS and vital signs were monitored simultaneously. The ability to self-report pain was also assessed in each patient immediately after the procedure. We aimed to test the inter-observer reliability and validity of the CPOT, the BIS and the combined use of these 2 instruments for pain detection in critically brain-injured patients. We hypothesized that the CPOT and the BIS could be used as reliable and valid instruments to detect pain in this population.

## 2 Methods

### 2.1 Ethics and informed consent

The study was approved by the Institutional Review Board of Beijing Tiantan Hospital, Capital Medical University, Beijing, China. Written informed consent was obtained from the patient or the appropriate substitute decision makers. The study protocol was retrospectively registered at ClinicalTrials.gov (NCT03368326).

### 2.2 Study design, settings, and participants

This prospective observational study was conducted in a 30-bed neurosurgical ICU in the Beijing Tiantan Hospital, Capital Medical University, Beijing, China. We included intubated and mechanically ventilated adult patients with brain injury, including traumatic brain injury, stroke (ischemic stroke, spontaneous intracerebral hemorrhage, and subarachnoid hemorrhage), intracranial operations for brain tumors, hypoxic-ischemic encephalopathy, and intracranial infection. The exclusion criteria were as follows: age <18 years, tetraplegia, epilepsy, agitation (i.e., Sedation-Agitation Scale ≥4),^[23]^ the use of muscle relaxants during the 24 hours prior to enrollment, pregnancy or lactation, moribund condition, and enrolled in another study related to analgesia and sedation. Patients were enrolled only once.

At each patient's entry into the study, data were collected on demographics, the type of brain injury, the motor response according to the Glasgow Coma Scale (GCS), the acute physiology and chronic health evaluation (APACHE) II score, the type of artificial airway and mode of ventilation, and the use and dosage of sedatives and analgesics.

### 2.3 Nociceptive and non-nociceptive stimulation

During the study, no attempts were made to change or influence the routine practice of patient care.^[24,25]^ After enrollment, 2 procedures were performed in a random crossover fashion, namely endotracheal suctioning (nociceptive stimulus) and gentle touching on the left shoulder (non-nociceptive stimulus). Endotracheal suctioning was a routine nursing procedure in mechanically ventilated patients in the ICU, and gentle touching was a standard component during the GCS evaluation in brain-injured patients.

Randomization was based on an online random number generator (http://www.psychicscience.org/random.aspx, accessed on January 1, 2016), and the patient allocations were sealed in numbered, opaque envelopes.

Closed suction catheters (12 F, Kimberly Clark, Carretera International Salida Norte, Sonora, Mexico) were used for the endotracheal suctioning. After 1-minute of preoxygenation with a 1.0 fraction of inspired oxygen, the suction catheter was introduced into the trachea at a depth of 15 cm. A suction pressure of –150 mmHg was applied continuously during the withdrawal of the catheter. The insertion and withdrawal of the catheter lasted approximately 15 seconds. Before the non-nociceptive stimulation (gentle touching), preoxygenation was also performed for 1 minute.

There was a 30-minute stabilization period before the first stimulation, and a 30-minute washout period between the 2 stimulations, during which time painful procedures, including suctioning, turning, venous or arterial catheterization, line and drain removal, and wound care, were avoided as much as possible. Adjustments of sedative and analgesic doses were also avoided.

### 2.4 Measurements

The CPOT was evaluated, and the BIS, heart rate (HR), and mean arterial pressure (MAP) were measured before and immediately after the stimulation.

The CPOT is composed of 4 behavioral domains, namely facial expression, body movements, muscle tension, and compliance with the ventilator in intubated patients (Supplemental Digital Content 1).^[26]^ Each item is rated on a 0 to 2 responsive score with a total score of 0 to 8. The Chinese version has been available since 2014^[27]^ and has been validated in adult ventilated patients without brain injury.^[27,28]^ Before the initiation of the formal study, 2 residents and 2 chief nurses were trained to use the CPOT in a 60-minute standardized session including both the Chinese and English version of the CPOT.^[26–28]^ The trainees also performed bedside assessments in 10 patients to guarantee complete comprehension of the tool. During the study, the 4 observers independently assessed each patient with the CPOT for 1 minute before and 1 minute immediately after the stimulation. For the endotracheal suctioning, the item “compliance with the ventilator” was assessed after the suction catheter was completely withdrawn from the endotracheal tube.

The BIS was recorded by a BIS A-2000 XP monitor (Aspect Medical Systems, Inc., Norwood, MA: Host version 3.31) with a Quattro Sensor (BIS^TM^ 4 Electrode Sensor, Aspect Medical Systems, Inc., Norwood, MA) attached to the left side of the forehead. The BIS monitor was connected to the sensor by a patient interface cable and a digital signal converter. The electrode impedance was maintained below 5000 Ω to ensure adequate signal quality. HR and non-invasive MAP were measured by a Philips IntelliVue MP60 patient monitor (Philips Electronics, Boeblingen, Germany). The maximum BIS and HR values during the 1-minute periods before and after the stimulation were documented.

After the completion of the post-stimulation CPOT assessment, the ability to self-report pain was evaluated by asking the patient “Are you in pain?” by another chief nurse who was blinded to the CPOT results. Whether the patient could respond to the question was documented. Thus, the patients were divided into 2 groups, namely those with and without the ability to self-report pain. Responsive patients could indicate the presence or absence of pain by nodding or shaking their heads.

### 2.5 Study endpoints

The study endpoints included:

(1) Inter-observer reliability of the CPOT;
(2) Criterion validities of the CPOT, the BIS, HR, and MAP in patients with the ability to self-report pain;
(3) Diagnostic accuracy of combining the CPOT and the BIS to detect pain;
(4) Discriminant validities of the CPOT, the BIS, HR, and MAP in all patients as well stratified by their ability to self-report pain.

### 2.6 Sample size

We used the discriminant validity of the CPOT in patients without the ability to self-report pain to estimate the sample size. Our previous study showed a standard deviation of 2 in the elevation of the CPOT score during endotracheal extubation.^[29]^ Thus, we needed to enroll 120 patients without the ability to self-report pain to have a Type I error (*α*) of 0.05 and a power (1–*β*) of 0.8, with a medium effect size of 0.5.^[30]^ Using the prevalence of 30% of neuro-critical patients who are non-responsive to the evaluation of the ability to self-report,^[31]^ we enrolled 400 patients in the present study. G-power software (version 3.1.9.3, Heinrich Heine University, Dusseldorf, Germany) was used for the sample size estimation.^[32]^

### 2.7 Statistical analysis

Categorical variables are presented as numbers and percentages. Continuous variables are presented as medians (25th to 75th percentile).

The inter-observer reliability of the CPOT was analyzed by the intraclass correlation coefficient (ICC), which was calculated from the assessments by the 4 observers (2 residents and 2 nurses) either before or after the stimulation as a “two-way random” model.^[33]^

In patients with the ability to self-report pain, the criterion validities of the CPOT, the BIS, HR, and MAP were tested using receiver operating characteristic (ROC) curve analysis. The areas under the curve (AUCs) and 95% confidence intervals (CIs) were calculated and compared between each pair of parameters.^[34]^ The cut-off value for each parameter was derived according to the simultaneous maximization of both the sensitivity and the specificity. The CPOT and the BIS were combined in 2 patterns to indicate pain as follows: “either way” as either CPOT ≥2 or BIS ≥88 after the stimulation and “both way” as both CPOT ≥2 and BIS ≥88 after the stimulation. The sensitivity, specificity, positive predictive value (PPV), and negative predictive value (NPV), as well as the respective 95% CIs, were reported.

For the discriminant validity analysis, the Scheirer–Ray–Hare test^[35]^ was used to compare the parameters before and after stimulation as well as between nociceptive and non-nociceptive stimulation. The Mann–Whitney *U* test was used to compare the change in the CPOT or the BIS after suctioning between patient with and without the ability to self-report pain.

The statistical analyses were performed with SPSS 17.0 (SPSS Inc., Chicago, IL) and MedCalc 17.9.7 (MedCalc Software, Ostend, Belgium). A *P* value of .05 was considered statistically significant.

## 3 Results

**Figure 1:**
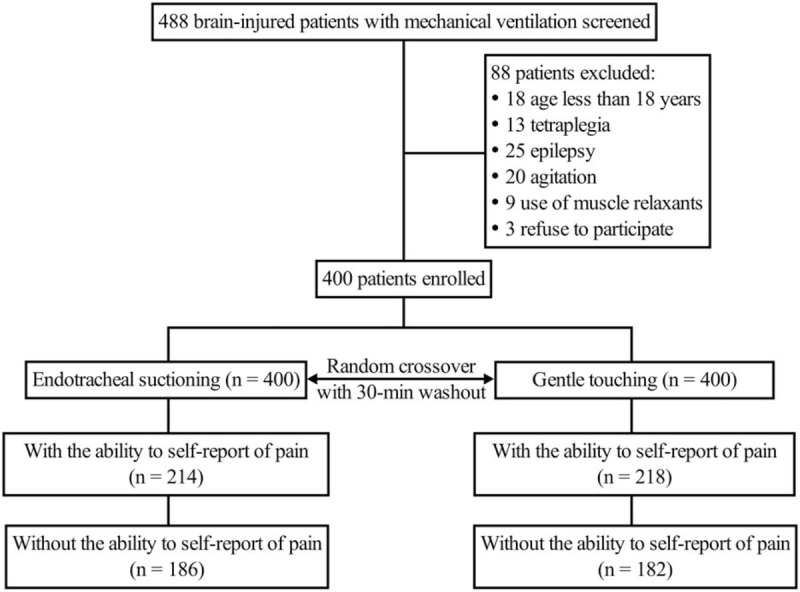
Patients flow chart.

From June 2015 to October 2017, we enrolled 400 brain-injured patients admitted to the ICU and undergoing mechanical ventilation (Fig. 1). The demographic and clinical characteristics of the patients at study entry are shown in Table 1. Most enrolled patients had undergone intracranial operations for brain tumors (212, 53%). Two procedures (endotracheal suctioning and gentle touching) were conducted in a random crossover manner in each patient. Endotracheal suctioning was first performed in 202 patients.

**Table 1:**
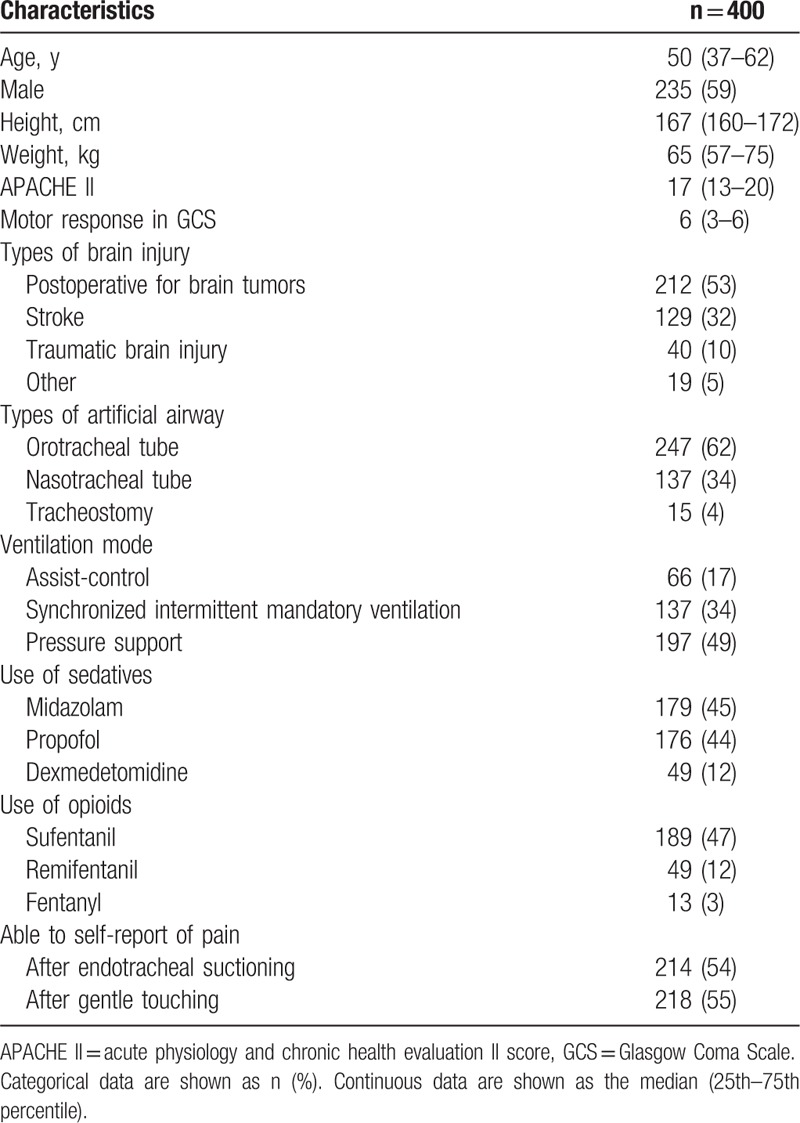
Demographic and clinical characteristics at the study entry.

The inter-observer reliability of the CPOT assessments among the 2 residents and the 2 nurses was nearly perfect, with the ICC (95% CI) ranging from 0.86 (0.83–0.89) to 0.93 (0.91–0.94) during the 2 stimulations and in all patients, as well as in patients stratified by their ability to self-report pain (Table 2). The CPOT results from the first nurse were used for further analysis.

**Table 2:**
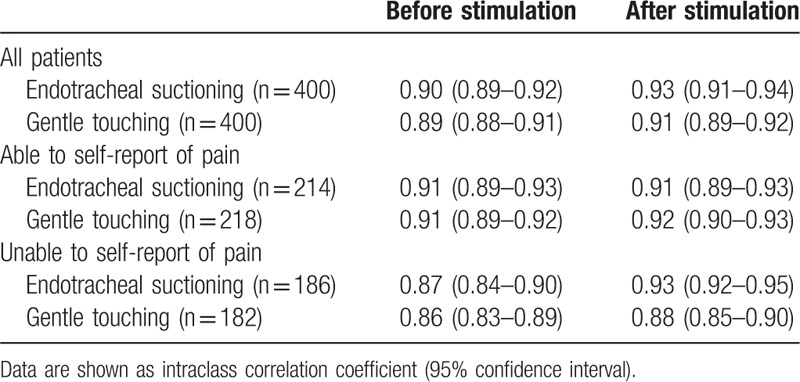
The inter-observer reliability of the critical-care pain observation tool assessments from 2 residents and 2 nurses.

**Figure 2:**
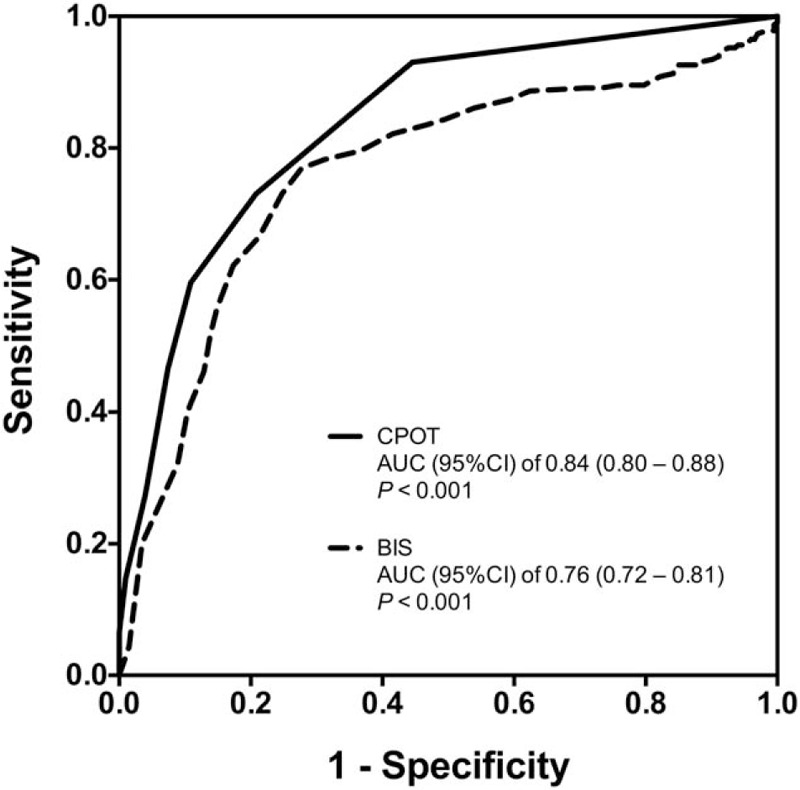
Receiver operating characteristic (ROC) curve for the critical-care pain observation tool (CPOT) and the bispectral index (BIS) for pain detection. The areas under the curve (AUCs) and 95% confidence intervals (CIs) are also shown. The AUC for the CPOT was significantly higher than that of the BIS (*P* = .01).

The ability to self-report pain was maintained in 214 (54%) and 218 (55%) patients during suctioning and gentle touching, respectively. There were 192 (90%) patients who reported pain after suctioning, while only 38 (17%) patients reported pain after gentle touching. Using the self-reported pain as the reference, the AUC (95% CI) for the CPOT (0.84 [0.80–0.88]) was significantly higher than that of the BIS (0.76 [0.72–0.81]) (*P* = .01, Fig. 2). The respective cut-off values for the CPOT and the BIS were 2 and 88. When the 2 instruments were combined as an “either way” (i.e., either CPOT ≥2 or BIS ≥88 after the procedure), the sensitivity, specificity, PPV and NPV were 0.90 (0.85–0.93), 0.59 (0.52–0.66), 0.72 (0.66–0.77), and 0.83 (0.76–0.89), respectively. While the 2 instruments were combined as a “both way” (i.e., both CPOT ≥2 and BIS ≥88 after the procedure), the respective sensitivity, specificity, PPV, and NPV were 0.62 (0.55–0.68), 0.89 (0.83–0.93), 0.86 (0.80–0.91), and 0.67 (0.61–0.73).

The AUCs (95% CIs) for HR and MAP were 0.64 (0.59–0.69) and 0.59 (0.53–0.64), respectively (Supplemental Digital Content 1, Figure S1). The cut-off value for HR was 101 beats/min, and the cut-off value for MAP was 99 mmHg. Data regarding the sensitivity and specificity (0.66 and 0.57 for HR, and 0.52 and 0.63 for MAP) are also shown in Supplemental Digital Content 2.

**Figure 3:**
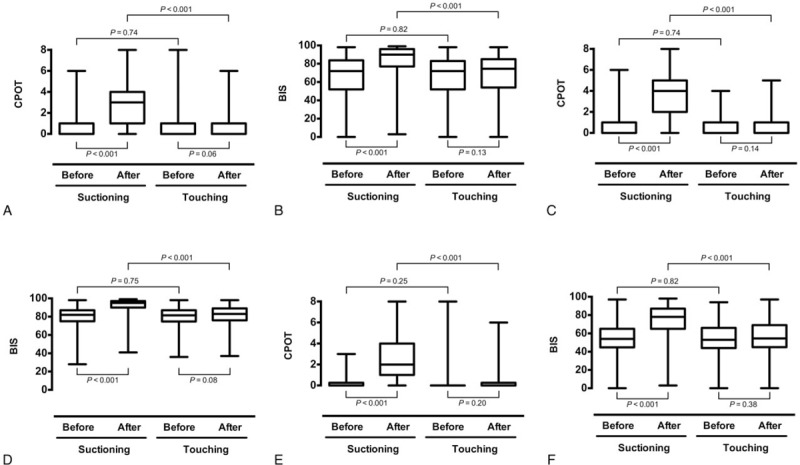
The discriminant validity of the critical-care pain observation tool (CPOT) and the bispectral index (BIS) in all patients (panels A and B), as well as in patients either with (panels C and D) or without (panels E and F) the ability to self-report pain. Pairwise comparisons are shown.

**Figure 4:**
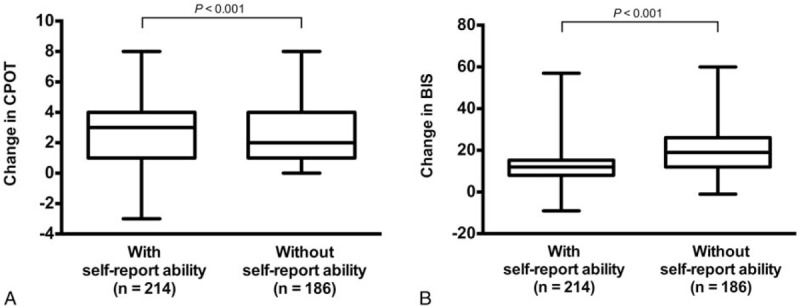
The changes in the critical-care pain observation tool (CPOT) and the bispectral index (BIS) after suctioning. The elevation of the CPOT (panel A) was significantly lower but the BIS elevation (panel B) was significantly higher in patients without the ability to self-report pain compared with the patients with the ability to self-report pain.

The CPOT and the BIS results before and after the 2 stimulations are shown in Fig. 3. In all patients, as well as in patients either with or without the ability to self-report pain, the CPOT and the BIS increased significantly after suctioning (all *P* < .001), while they remained unchanged after gentle touching (*P* ranging from .06 to .14). No significant differences were found in the pre-stimulation CPOT and BIS values between the 2 stimulations (*P* ranging from .74 to .82), but all post-stimulation values were significantly higher after suctioning than after touching (all *P* < .001). The changes in the CPOT and BIS values after suctioning are presented in Fig. 4. The elevation of the CPOT was significantly lower (2 [1–4] vs 3 [1–4]) but the BIS elevation was significantly higher (19 [12–26] vs 12 [8–15]) in patients without the ability to self-report pain compared with the patients with the ability to self-report pain (all *P* < .001).

Data regarding the discriminant validity for HR and MAP are shown in Supplemental Digital Content 3 (Figure S2 and S3). Although HR and MAP values increased significantly after suctioning (all *P* < .001), the elevation was only 8 (3–17) beats/min for HR and 7 (2–11) mmHg for MAP.

## 4 Discussion

In the present study, the results of the criterion and discriminant validities supported the use of the CPOT and the BIS for pain assessment in brain-injured patients undergoing mechanical ventilation. Combining use of the CPOT and the BIS might provide comprehensive pain assessment for different purposes. After the nociceptive procedure, the elevation of the BIS was more obvious in patients without the ability to report pain, suggesting that the BIS might be more suitable for pain screening in unconscious patients. We once again confirmed the previously reported inter-observer reliability of the CPOT.

During the study, several measures were taken to minimize the research bias. For the evaluation of pain self-reporting, we selected “yes or no” responses rather than a descriptive pain intensity scale (e.g., visual analogue scale or numerical rating scale) to indicate pain because many intubated and mechanically ventilated patients are unable to feasibly use pain intensity scales.^[26–28,36]^ Endotracheal suctioning and gentle touching are the 2 most commonly used stimuli in the analysis of the discriminant validity for pain indicators.^[15,18,20,21,28]^ We found that the incidence of self-reported pain was much higher after suctioning than after gentle touching (90% vs 17%), which also confirmed the discriminant capability for pain by these 2 stimuli. A 30-minute washout period was selected between the 2 stimulations because previous studies showed that the pain-induced elevations of hormones, for example, epinephrine and norepinephrine, were eliminated after this time interval.^[28,37]^ We did not collect respiratory rate as a potential pain indicator because a change of respiratory rate could be influenced by the endotracheal suctioning itself rather than the pain induced by the suctioning.

As a behavioral pain scale, the CPOT, developed by Gélinas et al^[38]^ in 2002 and 2003, has been validated in critically ill patients.^[15,20,21,26,28,36,39,40]^ Clinical guidelines also recommend the use of behavioral pain scales for monitoring pain in adult ICU patients without brain injury.^[1]^ The CPOT has also been assessed in brain-injured patients.^[16–19]^ Compared with non-nociceptive stimulation (non-invasive blood pressure cuff inflation or gentle touching), a significant increase in the CPOT was found during nociceptive stimulation (turning or mobilization for hygiene) in all 4 investigations, which indicated the discriminant validity of the CPOT.^[16–19]^ In patients with the ability to self-report pain, the CPOT positively correlated with pain intensity scales (numerical rating scale or Faces Pain Thermometer), and the AUCs in ROC analyses ranged from 0.72 (0.52–0.93)^[18]^ to 0.86 (0.76–0.97),^[17]^ which supported the criterion validity. Acceptable inter-rater reliability was also found in these studies. Our data confirmed these results with an AUC of 0.84 (0.80–0.88) (Fig. 2) and inter-observer ICCs ranging from 0.86 (0.83–0.89) to 0.93 (0.92–0.95) (Table 2), suggesting that the CPOT could be used as a valid and reliable monitoring tool for detecting procedure pain in critically brain-injured patients.

It is worth noting that behavioral pain scales may not be applicable in certain clinical situations and patient populations, such as those with reduced facial expressions and body movements due to drugs and diseases.^[15]^ It was also found that patients with traumatic brain injury might exhibit atypical behaviors during nociceptive stimulations.^[41]^ As a measurement of cortical activity, the BIS has been used to monitor the depth of general anesthesia.^[42]^ Preliminary studies showed that the BIS increased markedly during nociceptive procedures in critically ill patients, suggesting the BIS as a potential instrument for the detection of pain.^[20–22,43,44]^ In 25 traumatic brain-injured patients, Arbour et al^[22]^ demonstrated that BIS increased significantly during turning but not during blood pressure cuff inflation. Our ROC analysis in patients with the ability to self-report pain revealed an AUC of 0.76 (0.72–0.81) for detecting pain by the BIS (Fig. 2). We further tested the diagnostic accuracy after combining the use of CPOT and BIS in 2 patterns. A higher sensitivity was found for pain detection when the CPOT and the BIS were combined in an “either way” manner, but a higher specificity was found when the 2 instruments were combined in a “both way” manner. This might suggest different use of pain assessing instruments in different clinical situations. When pain screening is the main purpose, such as in patients with severe brain injury and those receiving hypothermic treatment, analgesia could be initiated as either an increase in the CPOT or an increase in the BIS after stimulation. On the other hand, when avoidance of the side effects of analgesics is the main consideration, such as in patients during the early postoperative period after intracranial operations, pain could be confirmed by the simultaneous elevation of the CPOT and the BIS. However, further investigation is needed for this combined use of CPOT and BIS.

Our data regarding BIS variations after suctioning and gentle touching supported the discriminant validity of the BIS (Fig. 3). Interestingly, the elevation of the BIS after nociceptive stimulation was much higher in patients without the ability to self-report pain (Fig. 4). This might result from a lower baseline BIS value in non-responsive patients and a larger margin for elevation after the stimulation. Thus, it seems that the BIS might be more suitable for pain assessment in patients with severely impaired consciousness, but further investigation is required.

In accordance with the clinical guidelines for non-brain-injured patients,^[1,2]^ studies in brain-injured patients also did not support the use of vital signs alone for pain assessment.^[45,46]^ In the present study, although statistical significances were found in HR and MAP, the sensitivity and specificity in the criterion validity test and the fluctuations in the discriminant validity test were of limited clinical significance (Supplemental Digital Content 2 and 3). Our results also suggested that vital signs should be used with caution.

There were limitations in the present study. First, we could not blind the observers to the type of stimulus during the procedure. As suctioning has been repeatedly demonstrated as one of the most painful ICU procedures,^[6,7]^ the observers might have tended to give a higher score after the suctioning because of the subjective nature of the CPOT. Second, we did not collect electromyogram data because this parameter cannot be obtained directly from the screen of the BIS monitor. It was shown that the facial electromyogram was also elevated during the nociceptive procedure but not during the non-nociceptive procedure,^[21,22]^ which might be explained by the increase in facial expressions due to pain. Additionally, we could not differentiate whether the elevation of BIS during suctioning was resulted from an increase in nociceptive stimulus and/or from a confounding effect of elevated electromyogram activities. Continuously digitizing the electromyogram may solve this problem and enhance its clinical use. Third, we enrolled a mixed brain-injured patient population with most of the patients being postoperative for brain tumors. Patients with different types of brain injury may exhibit different behaviors during painful stimulations. Further investigation is needed in patients with specific injury types.

In conclusion, the CPOT as well as the BIS seem to be reliable and valid instruments for pain assessment in critically brain-injured patients. The BIS may be more suitable for detecting pain in unconscious patients. Further validation of the combined use of CPOT and BIS is required in clinical situations with different purposes for pain assessment.

## Acknowledgments

The authors would like to thank Prof. Hong-Qiu Gu (Clinical Trial and Research Center, Beijing Tiantan Hospital, Capital Medical University, Beijing, China) for his valuable suggestions on statistical analysis. This work was supported by grants from the Beijing Health Bureau, Beijing, China (grant number 2014-2-2041). The sponsors had no role in the study design, data collection, data analysis, data interpretation, or writing of the report. The manuscript has been revised for grammar and spelling by the American Journal Experts.

## Author contributions

**Conceptualization:** Jing-Jing Hao, Jian-Xin Zhou.

**Data curation:** Kai Shan, Jing-Jing Hao, Xiu-Mei Sun, Yu-Mei Wang, Jian-Xin Zhou.

**Formal analysis:** Kai Shan, Xiu-Mei Sun, Yu-Mei Wang, Jian-Xin Zhou.

**Funding acquisition:** Jian-Xin Zhou.

**Investigation:** Kai Shan, Wei Cao, Yuan Yuan, Jing-Jing Hao, Xiu-Mei Sun, Xuan He, Gui-Yun Li, Yu-Mei Wang, Jian-Xin Zhou.

**Methodology:** Jian-Xin Zhou.

**Project administration:** Jian-Xin Zhou.

**Supervision:** Jian-Xin Zhou.

**Writing – original draft:** Kai Shan, Xiu-Mei Sun, Jian-Xin Zhou.

**Writing – review & editing:** Kai Shan, Wei Cao, Yuan Yuan, Jing-Jing Hao, Xiu-Mei Sun, Xuan He, Gui-Yun Li, Yu-Mei Wang, Jian-Xin Zhou.

## Supplementary Material

Supplemental Digital Content
