# Supplementary material for: Use of the critical-care pain observation tool and the bispectral index for the detection of pain in brain-injured patients undergoing mechanical ventilation: A STROBE-compliant observational study
Source: Medicine (Baltimore). 2018 Jun 1;97(22):e10985. doi: 10.1097/MD.0000000000010985 (PMC6392730; doi:10.1097/MD.0000000000010985)
Supplement: Supplemental Digital Content [file medi-97-e10985-s001.pdf]

Use of the Critical-Care Pain Observation Tool and the bispectral index for the detection of pain in brain-injured patients undergoing mechanical ventilation: a STROBE-compliant observational study

Kai Shan, Wei Cao, Yuan Yuan, Jing-Jing Hao, Xiu-Mei Sun, Xuan He, Gui-Yun Li, Yu-Mei Wang, Jian-Xin Zhou

<u>Supplemental Digital Content 2: Figure S1</u>

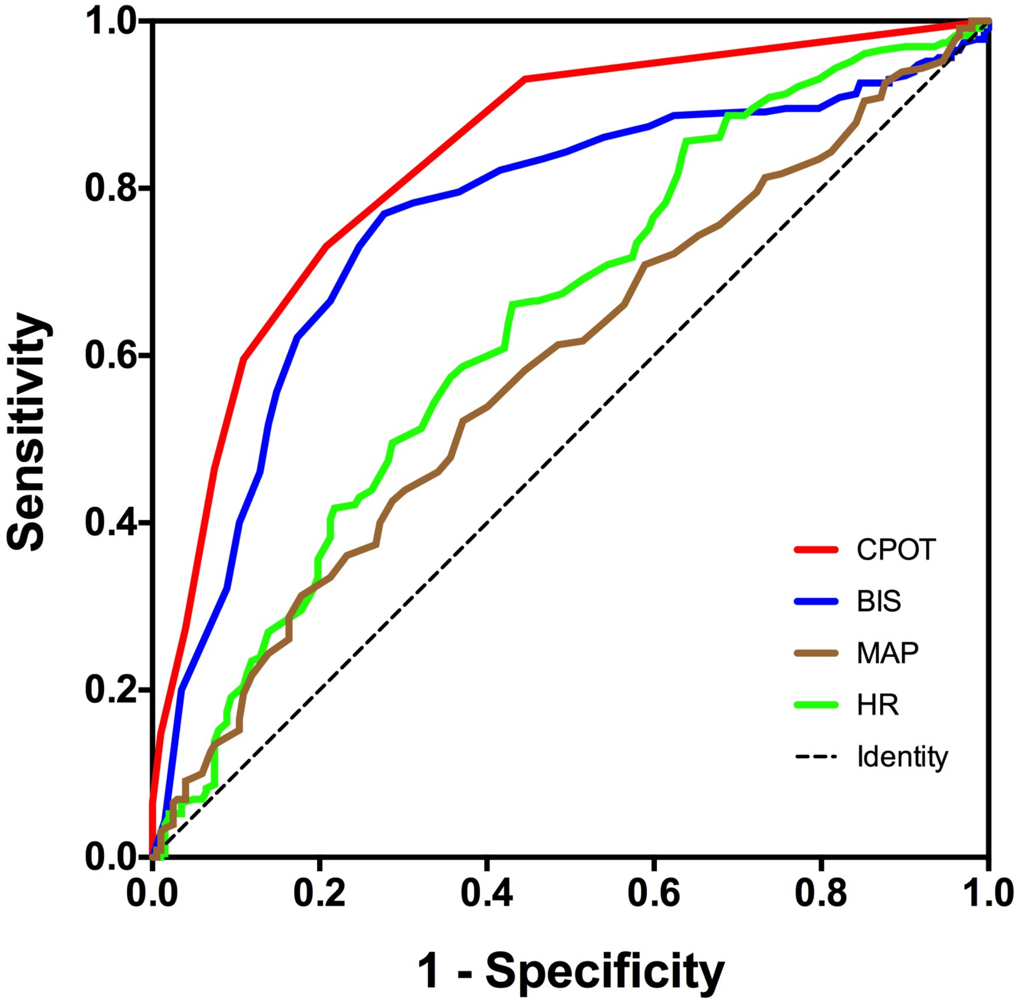

Figure S1. Receiver operating characteristic curve analysis in patients being able to self-report pain

Instruments	AUC (95% CI)	*P*	Cut-off value	Sensitivity	Specificity
CPOT	0.84 (0.80 – 0.88)	<0.001	2	0.73	0.79
BIS	0.76 (0.72 – 0.81)	<0.001	88	0.77	0.72
HR (beats/min)	0.64 (0.59 – 0.69)	<0.001	101	0.66	0.57
MAP (mmHg)	0.59 (0.53 – 0.64)	0.002	99	0.52	0.63

AUC = area under the curve, CI = confidence interval, BIS = bispectral index, CPOT = Critical-Care Pain Observation Tool, HR = heart rate, MAP = mean arterial pressure

Use of the Critical-Care Pain Observation Tool and the bispectral index for the detection of pain in brain-injured patients undergoing mechanical ventilation: a STROBE-compliant observational study

Kai Shan, Wei Cao, Yuan Yuan, Jing-Jing Hao, Xiu-Mei Sun, Xuan He, Gui-Yun Li, Yu-Mei Wang, Jian-Xin Zhou

Supplemental Digital Content 3: Figure S2 and Figure S3

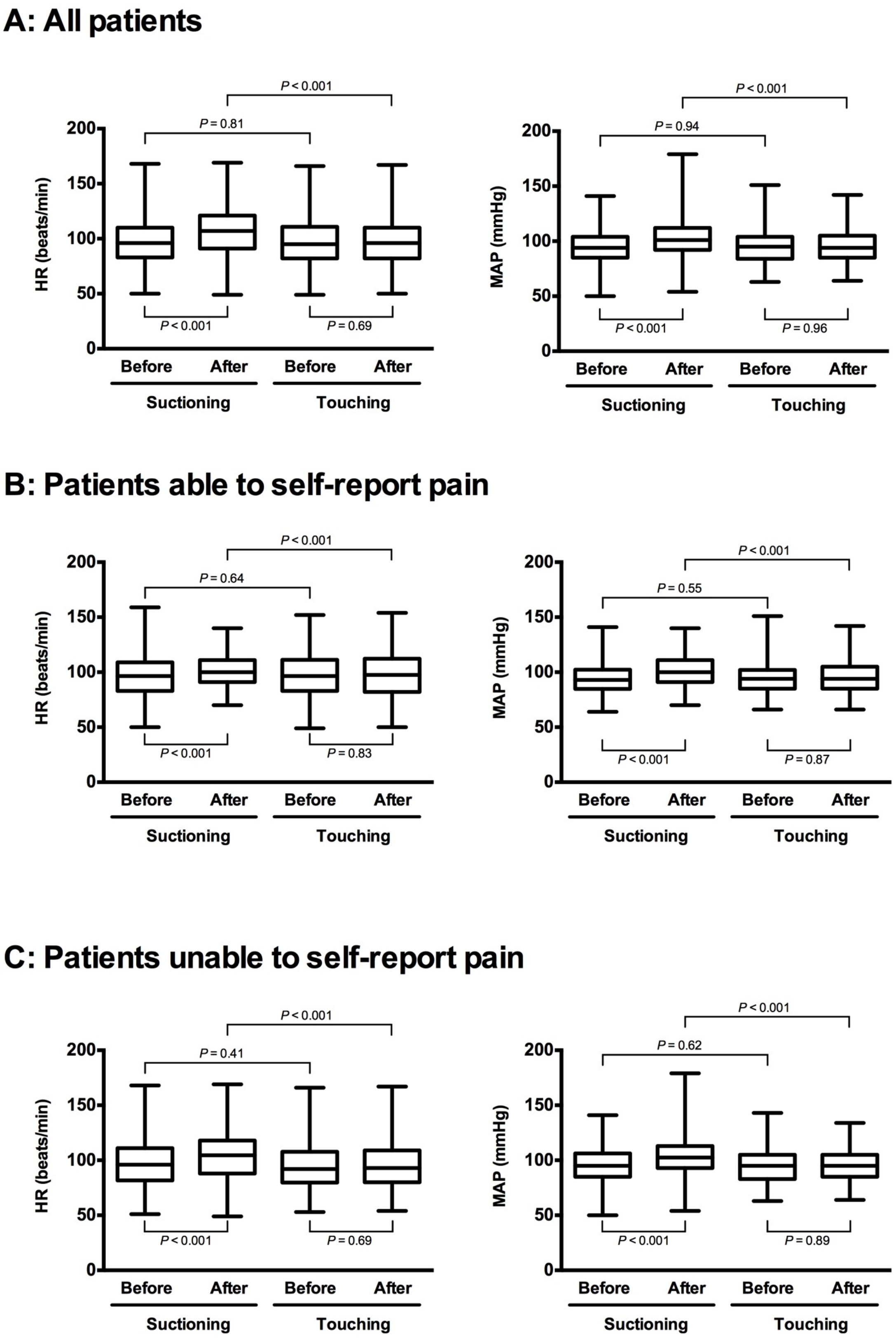

Figure S2. Heart rate (HR) and mean arterial pressure (MAP) before and after the two stimulations (endotracheal suctioning and gentle touching)

Pairwise comparisons are shown.

Use of the Critical-Care Pain Observation Tool and the bispectral index for the detection of pain in brain-injured patients undergoing mechanical ventilation: a STROBE-compliant observational study

Kai Shan, Wei Cao, Yuan Yuan, Jing-Jing Hao, Xiu-Mei Sun, Xuan He, Gui-Yun Li, Yu-Mei Wang, Jian-Xin Zhou

Supplemental Digital Content 4: Figure S3

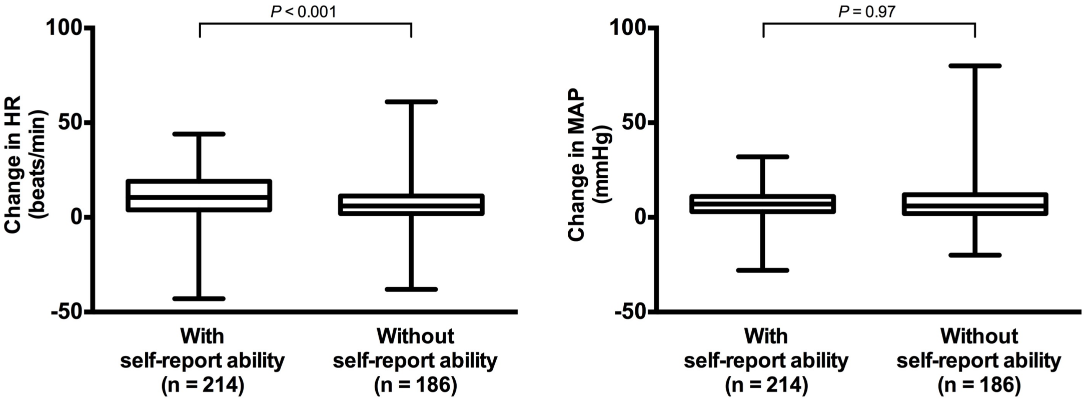

Figure S3. Changes in heart rate (HR) and mean arterial pressure (MAP) after suctioning in patients with and without the ability to self-report pain

Use of the Critical-Care Pain Observation Tool and the bispectral index for the detection of pain in brain-injured patients undergoing mechanical ventilation: a STROBE-compliant observational study

Kai Shan, Wei Cao, Yuan Yuan, Jing-Jing Hao, Xiu-Mei Sun, Xuan He, Gui-Yun Li, Yu-Mei Wang, Jian-Xin Zhou

Supplemental Digital Content 1: Table S1

Table S1. Description of the Critical-Care Pain Observation Tool

Indicator	Score		Description
Facial expression	Relaxed, neutral	0	No muscle tension observed
	Tense	1	Presence of frowning, brow lowering, orbit tightening and levator contraction or any other change (e.g. opening eyes or tearing during nociceptive procedures)
	Grimacing	2	All previous facial movements plus eyelid tightly closed (the patient may present with mouth open or biting the endotracheal tube)
Body movements	Absence of movements	0	Does not move at all (doesn't necessarily mean absence of pain) or normal position (movements not aimed toward the pain site or not made for the purpose of protection)
	Protection	1	Slow, cautious movements, touching or rubbing the pain site, seeking attention through movements
	Restlessness/Agitation	2	Pulling tube, attempting to sit up, moving limbs/thrashing, not following commands, striking at staff, trying to climb out of bed
Muscle tension	Relaxed	0	No resistance to passive movements
Evaluation by passive	Tense, rigid	1	Resistance to passive movements
flexion and extension of upper limbs when patient is at rest or evaluation when patient is being turned	Very tense or rigid	2	Strong resistance to passive movements or incapacity to complete them
Compliance with the ventilator	Tolerating ventilator or movement	0	Alarms not activated, easy ventilation
(intubated patients)	Coughing but tolerating	1	Coughing, alarms may be activated but stop spontaneously
or	Fighting ventilator	2	Asynchrony: blocking ventilation, alarms frequently activated
Vocalization (extubated patients)	Talking in normal tone or no sound	0	Talking in normal tone or no sound
	Sighing, moaning	1	Sighing, moaning
	Crying out, sobbing	2	Crying out, sobbing

The Critical-Care Pain Observation Tool is composed of four behavioral domains, namely facial expression, body movements, muscle tension and compliance with the ventilator in intubated patients. Each item is rated on a 0-2 responsive score with a total score of 0-8.
